# Simultaneous Detection and Identification of Enteric Viruses by PCR-Mass Assay

**DOI:** 10.1371/journal.pone.0042251

**Published:** 2012-08-01

**Authors:** Jingzi Piao, Jun Jiang, Bianli Xu, Xiaohong Wang, Yanfang Guan, Weili Wu, Licheng Liu, Yuan Zhang, Xueyong Huang, Pengzhi Wang, Jinyin Zhao, Xiaoping Kang, Hua Jiang, Yuanyin Cao, Yuling Zheng, Yongqiang Jiang, Yan Li, Yinhui Yang, Weijun Chen

**Affiliations:** 1 College of Plant Protection, Shenyang Agricultural University, Shenyang, China; 2 State Key Laboratory of Pathogen and Biosecurity, Institute of Microbiology and Epidemiology, Academy of Military Medical Sciences, Beijing, China; 3 Key Laboratory of Genome Sciences and Information, Beijing Institute of Genomics, Chinese Academy of Sciences, Beijing, China; 4 Center for Disease Control and Prevention of Henan Province, Zhengzhou, China; 5 Beijing Genomics Institute in Wuhan, Wuhan, China; 6 Affiliated Hospital of Academy of Military Medical Sciences, Beijing, China; University of Calgary & ProvLab Alberta, Canada

## Abstract

Simultaneous detection of enteric viruses that cause similar symptoms (e.g. hand, foot and mouth disease) is essential to the prevention of outbreaks and control of infections. In this study, a novel PCR-Mass assay combining multiplex polymerase chain reaction (PCR) with matrix-assisted laser desorption/ionization time-of-flight mass spectrometry (MALDI-TOF MS) was developed and used for simultaneous detection of eight distinct human enteric viruses. Enteric viral isolates and standard viral RNAs were examined to determine the sensitivity and specificity of the PCR-Mass assay. Clinical performance was evaluated with a total of 101 clinical specimens from patients suspected of having hand, foot and mouth disease (HFMD). The results were compared to those of previous analyses using real-time RT-PCR. The identification of specific viruses and clinical specimens shows that the PCR-Mass assay performed as well as or better than standard methods with respect to indicating the presence of multiplex pathogens in a single specimen.

## Introduction

Simultaneous detection and classification of all potentially infectious pathogens in a given sample, even when the pathogens cause similar signs and symptoms, is essential to providing the true pathogen spectrum. Clinical syndromes are seldom specific to a single pathogen, so accurate and prompt identification of multiplex pathogens has proven invaluable not only to further treatment of the illness, but also to the control of infectious outbreaks and the efficient use of antibiotics and antiviral drugs [1].

The manifestations of human enteric viral infections, which are the most common illnesses during early childhood, range from gastroenteritis to life-threatening diseases such as hand, foot, and mouth disease (HFMD) [2], [3]. Different species of viruses, including enteroviruses, noroviruses, and astroviruses, have been known to be associated with these diseases. Despite the close genetic relationship between some enteric viruses, enterovirus 71 (EV71) is generally known to be the major cause of neurological complications and even fatalities in the Asia-Pacific region [4]–[6]. Most confirmed cases of EV71-associated HFMD have initial symptoms similar to those of other enteric viral infections but, more frequently, they cause severe neurological complications, including aseptic meningitis, brainstem encephalitis, and acute neurogenic pulmonary edema, none of which are characteristic of enteric viral infection. Accurate identification of human enteric viruses and early diagnosis from clinical specimens is crucial to patient management and control of infection.

Classical detection of enteric virus is mostly based on conventional approaches, including immunohistochemical detection, indirect immunofluorescence assay (IFA), cell culture, and neutralization testing. These immunological methods are restricted by the number of pathogens they can detect. Many clinical samples remain undetectable due to the absence of antibodies against viral surface proteins. Cell culture and neutralization tests, however, are time-consuming and laborious. Several studies have investigated the capability of multiplex RT-PCR and real-time PCR assays to diagnose enteric virus infections, and they showed good sensitivity and specificity [7], [8]. However, these methods are limited with respect to the number of target pathogens that can be effectively detected out of a multiplex assay. Misdiagnosis might be frequent in clinical cases. A more efficient method of diagnosing broad-spectrum enteric viruses in complex clinical specimens is needed.

The MALDI-TOF MS system is a high-throughput technology with multiplex capacity. It has already been used for genotyping [9]–[11]. Recently, it has been developed into a means of detecting viruses, including respiratory pathogens and human papillomavirus (HPV) [12]–[14]. As an alternative, the MALDI-TOF MS assay has the advantages of high throughput and short turnaround time. The main advantage of the MALDI-TOF MS is that it measures the intrinsic physical properties of molecules directly, while other methods are dependent on indirect detection of fluorescent or radioactive reporter tags [15]–[17].

In the present study, a novel PCR-Mass assay combining multiplex PCR with MALDI-TOF MS technology was developed for the simultaneously detection of eight distinct human enteric viruses, including poliovirus (PLV), coxsackievirus A16 (CoxA16), enterovirus 71 (EV71), hepatitis E virus (HEV), echovirus (ECHO), norovirus (NVG), astrovirus (ASTRV), and reovirus (REV). This study represents the first use of the PCR-Mass assay on enteric viruses in clinical specimens and its first direct comparison to currently implemented methods of detection.

## Materials and Methods

### Ethics Statement

All stool specimens were collected by the Center for Disease Control and Prevention of Henan Province. The link provided in the manuscript includes the HFMD diagnostic criteria (http://www.moh.gov.cn/publicfiles/business/htmlfiles/mohyzs/s3586/201004/46884.htm). Section one lists clinical manifestations, and sections four and five give diagnostic criteria. Based on this guideline, HFMD patients were diagnosed mainly by observation of the presence of vesicles on the palmar and plantar skin, buccal mucosa, and tongue. Medical practitioners collected stool samples from each suspected patient at the hospital using routine clinical laboratory procedures.

This study was approved by the Review Board of the Academy of Military Medical Sciences and the Affiliated Hospital, Center for Disease Control and Prevention of Henan Province, Beijing Institute of Genomics, and BGI in Wuhan. All subjects provided written informed consent.

### Virus Strains and Clinical Samples

Our study used a panel of human enteric viruses and other medically relevant organisms to assess the analytical specificity of PCR-Mass. PLV (strain Sabin 1), CoxA16 (strain shzh99-6), EV71 (strain 605/SHENZHEN/08/China/HFMD Severe/2008), and REV (strain Lang) were obtained from the Institute of Microbiology and Epidemiology (Academy of Military Medical Sciences, China). HEV (isolate HE-JI4), ECHO (strain Gregory), NVG5 (strain Hu/GII-4/Saga1/2006/JP) and ASTRV (strain Beijing/293/2007/CHN) were obtained from the Beijing Institute of Genomics (Chinese Academy of Sciences, China). The other medically relevant organisms (*Bacillus cereus, Salmonella enteritidis,* and *Streptococcus pneumoniae*) were gifted from the National Institutes for Food and Drug Control (Beijing, China).

From April 2009 through October 2009, clinical stool specimens from 101 suspected cases of HFMD were collected from the Center for Disease Control and Prevention of Henan Province. These specimens tested positive for enterovirus by RT-PCR. Stool specimens were obtained by trained staff according to diagnostic criteria set forth by the Ministry of Health (http://www.moh.gov.cn/publicfiles/business/htmlfiles/mohyzs/s3586/201004/46884.htm). These samples were collected from patients showing HFMD symptoms. Stool samples were also collected from 40 healthy people who had tested negative for enterovirus infection by RT-PCR. All samples were frozen at −80°C prior to further study.

Sample preparation, RNA extraction, PCR-Mass assay and real-time RT-PCR were conducted in different rooms. Special care was taken to avoid both contamination with RNase and cross-contamination between reactions.

### Sample Preparation

The stool suspension was made by adding 0.5 g of stool (0.5 ml for fluid stools) to 5 ml of 1×phosphate-buffered saline (PBS) with 10% chloroform. The suspension was then centrifuged at 6000 rpm/min for 15 min. the supernatant was then collected for the following tests.

### DNA and RNA Extraction

DNA and RNA were extracted from 200 µL of each sample using a TIANamp virus DNA/RNA kit (Tiangen Biotech) according to the manufacturer’s instructions.

### Standard Viral RNA

Viral target genes obtained by reverse transcription of RNA extracts or by synthesized plasmids that were assembled of polynucleotide fragments, were amplified by PCR. The PCR products were ligated with pGET-T Easy vector (Promega). RNA extracts and plasmids solutions were quantified spectrophotometrically at 260 nm and diluted to tenfold serial dilution. This yielded viral copies ranging from 1×10^0^ copies to 1×10^9^copies [18].

### Design of PCR Primers and Extension Probes

Virus sequences were obtained from the GenBank database. Then multiple sequence alignment was performed using the ClustalX 2.0 multiple sequence alignment program to select the most highly conserved regions.

Multiplex PCR primers and extension probes were selected from these conserved regions and designed using MassARRAY assay design software (Sequenom). Primers for multiplex PCR amplification were designed for both sides of the most conserved regions, including the extension probes. Optimal amplicon size was set at 80 to 120 bp in length for convenience of subsequent extension reactions. The amplicons covered the sequences of extension probes, each between 18 and 24 nucleotides long (approximately 5000 to 8500 Da). For best results, the mass of the amplification primer should be different from that of the extension probe and its extension products. The mass range of MS analytes is 5000 to 8500 Da. In order to ensure the positive results of EV71, CoxA16, and ECHO, a set of EV71-CMN primers and probe was added in the assay. These primers and probe were designed for conserved regions of the universal enteroviruses. All primers and probes were synthesized by Shanghai Sangon Company (Shanghai, China). The multiplex PCR primers and extension probes are listed in Table 1.

**Table 1:** Primers and probes used for the PCR-Mass assay.

Virus	Primers	Sequence (5′-3′)	Amplicon size (bp)	Probe
Echovirus	Forward	TGGGACGCTTCAATACTGACATGG	88	TGCGAAGAGTCTATTGAGCT
	Reverse	CAGTTAGGATTAGCCGCATTCA		
Coxsackievirus A16	Forward	GGTGCAGAAACACCATCATC	100	CAAACCTGGTACCAATCAGT
	Reverse	GCAGTAAAGCAAGGCATACCA		
Astrovirus	Forward	GGAATTCTGATATCCGGCAGA	81	ACCCCATTTACACCAGGATA
	Reverse	CATTCTGATGCGCCTCTAAG		
Reovirus	Forward	TTGGATCTCTGCAACGCAAG	98	TGAAGCATTTGCCTCACCATA
	Reverse	CGACGTAATCCTGATGACGA		
Enterovirus 71	Forward	ACCCTATCTCCCTGGATGGT	103	AAGTTGTGCAAGGATGCTAG
	Reverse	GCAGCCCAAAAGAACTTCAC		
Norovirus	Forward	GTCCCTTGACAAGACCACTTC	80	CACATGCGGAAAAACGAC
	Reverse	CTGTGAAGGATTCCCCGTT		
Hepatitis E virus	Forward	TAAAGCTTCACTGTCGGCTC	103	AGACGACGGGGCGAGAGTAAAATA
	Reverse	GAATTGATTTCGTCGGCTGG		
Poliovirus	Forward	ACGTTCACTCCTGACGACAA	112	CTCCTCCTGAGCGCAAGTACTCC
	Reverse	TGCCATTTCCAAAGAGGTAATCC		

### PCR-Mass Array

cDNA was reverse-transcribed using PrimeScript RT-PCR Kit (TaKaRa) according to the manufacturer’s protocols. In brief, the cDNA was synthesized by reverse transcription from 2 µL of RNA in a 20 µL solution containing 1 µL dNTP mixture (10 mM each), 1 µL random primer, 4 µL 5×PrimeScript Buffer, 0.5 µL RNase Inhibitor, 0.5 µL PrimeScript RTase. cDNA synthesis was performed at 42°C for 30 min, and the reaction was stopped by heating at 95°C for 5 min. Each PCR reaction contained 500 nM of each primer, 500 µM dNTP, 0.5U Hot Star Taq DNA polymerase (Qiagen Inc., Hilden, Germany), 25 mM MgCl_2_, 1 µL template DNA or 1 µL cDNA, and buffer supplied with the enzyme (final concentration 1x) to a final reaction volume of 5 µL. Thermocycling began with a denaturation step at 94°C for 15 min, followed by 45 cycles at a melting temperature of 94°C for 20 s, an annealing step at 56°C for 30 s, an extension step at 72°C for 1 min, and finally an extension step at 72°C for 3 min. In order to neutralize unincorporated dNTPs and render them unavailable to future extension reaction, the PCR product was then dephosphorylated with shrimp alkaline phosphatase (SAP). Next, 7 µL of SAP product was added to 2 µL of extension mix. Each extension reaction contained 0.2 µL Termination mix, 0.94 µL Extend Primer mix, 0.041 µL enzyme, 0.2 µL Buffer Plus (10x), and 0.619 µL of H_2_O to a complete volume of 2 µL. The extension mixture was thermocycled using the following parameters: I) denaturation at 94°C for 30 s, II) denaturation at 94°C for 5 s, III) annealing at 52°C for 5 s, IV) extension at 80°C for 5 s, V) go to III) 4 more cycles, VI) go to II) 39 more cycles, and VII) 72°C for 3 min. In the mixture, all four mass modified nucleotides (A, T, C, and G) was present. During the reaction, the probe was extended by one of the nucleotides, these terminated the extension of the probe. Each present virus can be analyzed using the extension products of different masses. The unextended probes indicated the absence of each virus. The extension products and calculated masses are provided in Table 2.

**Table 2:** Extension products of the PCR-Mass assay.

Virus	Peak description	Length of product (bp)	Calculated mass (Da)
ECHO	Extension Probe	20	6172.0
	Extension Probe + A	21	6443.2
coxA16	Extension Probe	20	6070.0
	Extension Probe + T	21	6397.1
astrv	Extension Probe	20	6030.0
	Extension Probe + G	21	6317.2
REV	Extension Probe	21	6365.2
	Extension Probe + G	22	6652.4
EV71	Extension Probe	20	6221.0
	Extension Probe + T	21	6548.1
NVG5	Extension Probe	18	5510.6
	Extension Probe + T	19	5837.7
HEV	Extension Probe	24	7508.9
	Extension Probe + C	25	7796.1
PLV	Extension Probe	18	5444.6
	Extension Probe + G	19	5731.8

After desalting by the addition of 6 mg clean resin, the optimized extension products were analyzed by MALDI-TOF MS spectrometer. Spectral data were interpreted using MassARRAY Typer Analyzer software (Sequenom). Human β-globin gene (HBB) and distilled water were added to the amplification mix as internal and negative controls. All extension system and Mass spectra contained within these reactions were obtained from Sequenom and used according to the manufacturer’s protocols.

### Real-time RT-PCR Methods for EV71 and CoxA16

All specimens were analyzed by real-time RT-PCR for EV71 and CocA16. Samples that showed discrepancies between the PCR-Mass and real-time RT-PCR were reanalyzed by sequencing. In addition to the aforementioned virus, six more enteric viruses, including ECHO, ASTRV, REV, NVG, HEV, and PLV were also detected by PCR-Mass. Results for the latter viruses were excluded from the comparison. Real-time RT-PCR of EV71 and CoxA16 was performed according to the published method [19]. Real-time RT-PCR was performed in a 30 µL mixture containing 6 µL of the RNA, 15 µL 2×Taqman one-step RT-PCR Master Mix (ABI, 4309169), 0.75 µL 40×MultiScribe and RNase inhibitor mixture, 0.25 µM of forward primer, 0.25 µM of reverse primer, and 0.2 µM of probe in a fluorometric PCR instrument (ABI 7300). The reaction was carried out for 30 min at 48°C followed by 10 min at 95°C and another 40 cycles of amplification (95°C for 15 s; 60°C for 40 s. Fluorescence FAM was recorded at 60°C).

**Figure 1:**
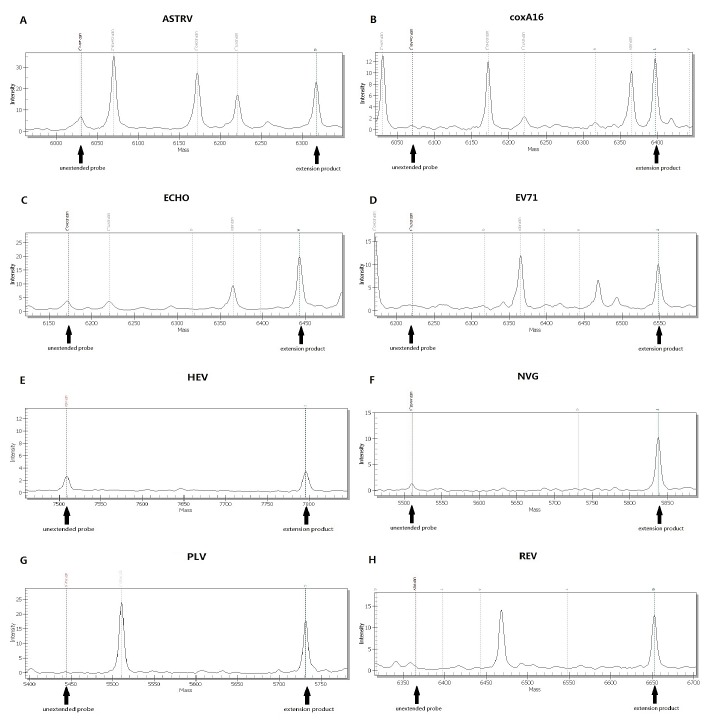
Mass spectra of different enteric viruses detected by PCR-Mass. (A) ASTRV; (B) coxA16; (C) ECHO; (D) EV71; (E) HEV; (F) NVG5; (G) PLV; (H) REV.

### Virus Isolation

Human rhabdomyosarcoma (RD) cell lines were inoculated with 101 stool samples from patients. Each sample was subjected to at least three cell culture passages in RD cells before being considered negative. The medium was replenished on day 7, and cultures were terminated 14 days after inoculation. All cultures were observed daily for cytopathic effects (CPE). All virus-infected cells were examined by RT-PCR for EV and by real-time RT-PCR for EV71 and CoxA16 at each passage.

**Figure 2:**
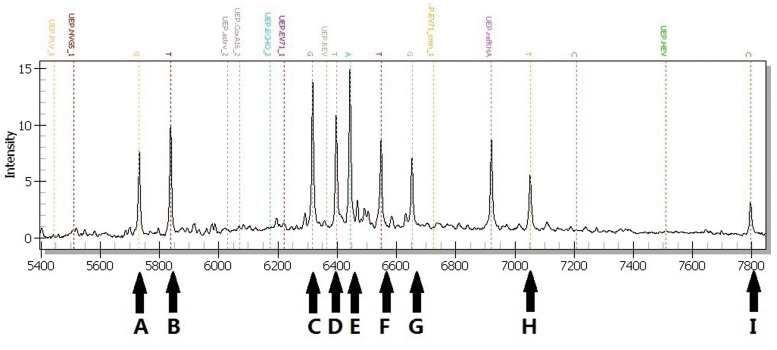
Specificity testing of the PCR-Mass assay with multiple pathogens. Eight human enteric viruses in the simulated sample were detected: A, PLV; B, NVG; C, ASTRV; D, coxA16; E, ECHO; F, EV71; G, REV; H, EV71-CMN; I, HEV.

## Results

### Specificity of the PCR-Mass Assay

In order to evaluate the efficacy of the primers and probes used in the PCR-Mass assay, we tested various reference strains of human enteric viruses. All of the reference strains were accurately identified by their specific probes and showed no significant cross-reaction to other probes, as shown in Figure 1. For the negative controls, no extension probe was demonstrated. This result suggests that the primers and probes are specific to their respective reference enteric viruses.

Then the various combinations (3 to 8) of virus templates were used to evaluate the ability of the PCR-Mass assay to identify multiple pathogens. As shown in Figure 2, the extension probes effectively detected all eight distinct viruses. These results indicate that PCR-Mass is an effective means of detecting multiple viruses with high specificity, even when as many as eight viruses are present.

In contrast, for other medically important pathogens, including *Bacillus cereus, Salmonella enteritidis,* and *Streptococcus pneumoniae,* we did not detect any extension for the probes. We did observe extension of internal control probes. All of the stools samples from our 40 healthy people tested negative.

### Sensitivity of the PCR-Mass Assay

Determination of detection limit and analytical sensitivity of the PCR-Mass assay was based on serial tenfold diluted standard viral RNAs by the theoretical number of copies. The results showed that the PCR-Mass assay to have a sensitivity of 100 copies per reaction for EV71, CoxA16, REV, PLV and EV71-CMN and a detection limit of 1000 copies per reaction for ECHO, ASTRV, NVG5 and HEV.

**Table 3:** Comparison of the PCR-Mass assay with real-time RT-PCR and sequencing methods for detection of enterovirus 71 and coxsackievirus A16 in clinical specimens.

Result by PCR-Mass	No. of specimens testing by real-time RT-PCR (no. of specimens retested by sequencing)			
	EV71		coxA16	
	Positive	Negative	Positive	Negative
Positive	84	4 (2)^a^	11	1 (1)^b^
Negative	3 (0)^c^	10	6 (0)^d^	83
Sensitivity (%)	100%		100%	
Specificity (%)	83.3%		98.8%	
Overall agreement (%)	93.1%		93.1%	

a Sequencing validated the EV71 positive for two of the four samples but invalidated for other two samples.

b One sample was confirmed as coxA16 negative by sequencing.

c Three samples initially detected as EV71 negative by PCR-Mass were confirmed as negative by sequencing.

d Six samples initially detected as coxA16 negative by PCR-Mass were confirmed as negative by sequencing.

### Detection of Clinical Specimens by Viral Isolation

There were 73 samples that tested positive by culturing methods. Initial CPE analysis showed rounded refractile cells 2–4 days after inoculation. All 73 isolates tested positive for enteroviruses by real-time RT-PCR. Out of 73 isolates, 60 isolates showed the presence of EV71 RNA, 5 isolates showed the presence of CoxA16 RNA, 3 isolates showed the presence of EV71 and CoxA16 RNA, and 5 isolates showed no EV71 or CoxA16 RNA (refer to Figure S1).

### Detection of Clinical Specimens by Real-time RT-PCR

Out of 101 stool samples containing universal enteroviruses, 11 specimens tested positive for CoxA16, 81 specimens tested positive for EV71, 6 specimens tested positive for EV71 and CoxA16, 3 specimens tested negative for EV71 and CoxA16.

### Detection of Clinical Specimens Using the PCR-Mass Assay

A total of 101 clinical stool samples had been found to be positive for enteroviruses under RT-PCR, but they had not been tested for any other pathogen. Samples that had tested negative for human enteric viruses were also included for testing as control.

By using PCR-Mass assay, 97/101 (96.0%) specimens were confirmed as positive for enteric viruses. The result showed 88/101 (87.1%) EV71 samples, 12/101 (11.9%) CoxA16 samples, 27/101 (26.7%) ECHO samples, 3/101 (3.0%) HEV samples, and 4/101 (4.0%) samples to be negative. For both EV71 and coxA16 virus, the PCR-Mass assay provided an overall agreement of 93.1%. (Table 3).

For the disagreement specimens, direct sequencing was conducted to confirm the results. Sequencing confirmed that 9 samples identified as false-negative samples under PCR-Mass were in fact negative (3 EV71, and 6 coxA16). This suggests that the samples no longer contained any detectable viral material. This may have been due to degradation of the RNA during storage or transport. Sequencing also confirmed that 2 EV71 false-positive samples were indeed positive, but it found 3 other samples to be negative (2 EV71, and 1 CoxA16).

The PCR-Mass assay showed a detection sensitivity of 100% and a specificity of 83.3% for EV71 virus. For coxA16 virus, the sensitivity and specificity were 100% and 98.8% respectively. Overall, the PCR-Mass results agreed with direct sequencing more closely than real-time RT-PCR did.

In addition to the detection of single virus in the clinical samples, the assay also detected several multi-infected samples, primarily possible co-infections. The presence of multiple pathogens was verified in a subset of 32 clinical samples by using PCR-Mass. Out of the 32 co-infections, 29 involved two viruses, and 3 involved three viruses. (Table 4).

**Table 4:** Co-infections detected by the PCR-Mass assay in clinical specimens.

No. of viruses detected(No. of co-infected specimens)	Viruses detected	No. of clinical specimenswith virus combination
Two(29)	EV71+coxA16	4
	EV71+ECHO	20
	EV71+HEV	1
	ECHO+coxA16	4
Three(3)	EV71+ECHO+HEV	2
	EV71+ECHO+coxA16	1

For 25 samples identified as ECHO by PCR-Mass, 17 cases were identified as ECHO using published species-specific PCR primers, and 11 cases were confirmed as ECHO in tests that used sequencing (data not shown). The lack of samples makes it difficult to properly estimate the clinical sensitivity and specificity for some of the pathogens detected by PCR-Mass, and so data related to these pathogens, such as HEV, are not reported here.

Together, these assay results suggest that the enteric virus diagnostic PCR-Mass assay can provide the necessary sensitivity and specificity for the detection of EV71, CoxA16 and other enteric viruses in clinical and laboratory settings.

## Discussion

The PCR-Mass assay used multiplex PCR conjugated MALDI-TOF MS technology to solve the intrinsic difficulties in simultaneous detection of enteric viruses. The novel assay used pairs of carefully designed multiplex PCR amplification primers with extension probes to prevent false positives without sacrificing sensitivity.

The detection of standard viral RNAs indicated that the assay has sensitivity levels ranging from 100 to 1000 copies/reaction. The study also shows that the assay can be used effectively for the simultaneous identification of complex co-infections of as many as eight viruses in simulated samples. Further testing using a total of 101 clinical stool specimens collected from patients suspected of having HFMD demonstrated that PCR-Mass results had better agreement with direct sequencing than real-time RT-PCR did.

Unlike the current standard method, PCR-Mass was generally able to detect a theoretical single copy of viral RNA. Real-time RT-PCR was much less sensitive, even when performed on fresh specimens. PCR-Mass was performed on specimens that had been frozen and thawed. Discrepancies for specific viruses within some samples may have been due to RNA degradation during storage or transport.

**Figure 3:**
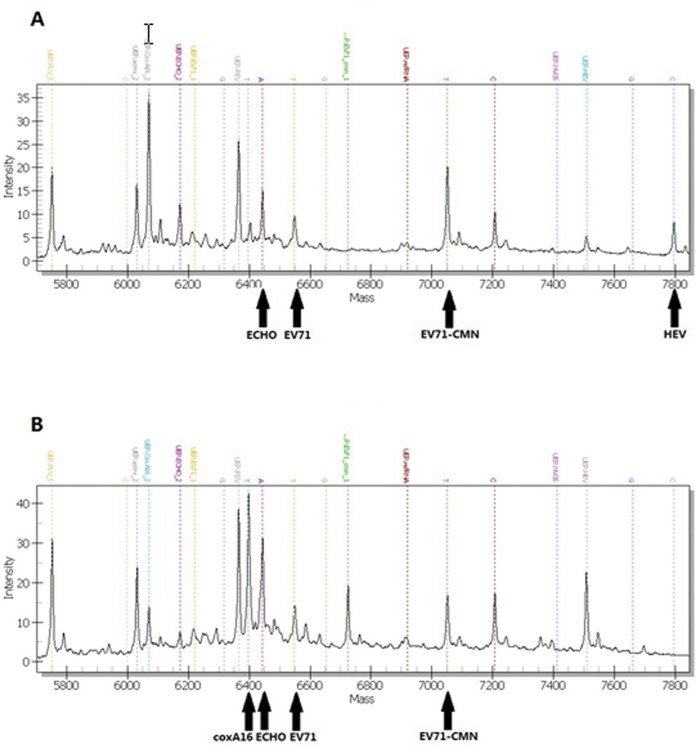
Simultaneous detection of clinical specimens. EV71-CMN was added as positive control. (A) Sample positive for EV71, ECHO, and HEV; (B) Sample positive for EV71, ECHO, and coxA16.

In addition to its main advantages, such as sensitivity and specificity, the PCR-Mass assay can also be used to identify viruses in co-infected samples. During our clinical study, we identified 32 specimens co-infected with two or three viruses (Figure 3). Although HFMD is frequently caused by EV71 and coxA16, there are increasing numbers of reports of other viruses associated with HFMD outbreak or sporadic cases [20], [21]. Infection with a single virus rarely results in severe cases, but co-infection was associated with severe clinical syndrome with rapid progressive neurological and cardiopulmonary complications [22]–[24]. In these cases, infection with other enteric viruses can be overshadowed by the major virus. This can cause the risks of developing more severe illness, complications, or fatalities to be underestimated. Multiplex assays, such as PCR-Mass, which are capable of simultaneous detection of multiple pathogens in a single assay can prevent misdiagnosis and delays in treatment without increasing costs or adding a step to the process.

While the PCR-Mass assay can be used to perform specific, sensitive, and simultaneous detection for enteric viruses, it is still a novel method of detection and pathogen coverage remains incomplete. The major limitation of the PCR-Mass assay is the design of the primers and probes. Each new assay requires recalibrating the mix of multiplex primers and probes. In our ongoing work, we will attempt to simplify the redesign method and try to develop a form of the assay that has more complete pathogen coverage. In its current form, the assay can be performed at a diagnostic laboratory in 7 h when partially automated, so future development will include decreasing the assay time, the development of full automation, and the reduction of the possibility of human error.

In this study, we report the development and extensive validation of a potentially high-throughput novel PCR-Mass assay. This assay can be used to simultaneously detect up to eight distinct viruses associated with enteric infections. This assay uses a mixture of amplifying primers and extension probes. It matched or exceeded the sensitivity and specificity of current methods while still maintaining coverage of a wide range of enteric viruses that would otherwise have been missed. Based on our results, we believe that this assay may serve as an alternative method for the improvement of clinical diagnosis of co-infections in surveillance and diagnostic applications.

## Supporting Information

Figure S1 **The results of viral isolation.** (A) negative control. (B) CPE of EV71. (C) CPE of CoxA16.
(TIF)
