# Supplementary figures and images for: Simultaneous Detection and Identification of Enteric Viruses by PCR-Mass Assay
Source: PLoS One. 2012 Aug 1;7(8):e42251. doi: 10.1371/journal.pone.0042251 (PMC3411642; doi:10.1371/journal.pone.0042251)

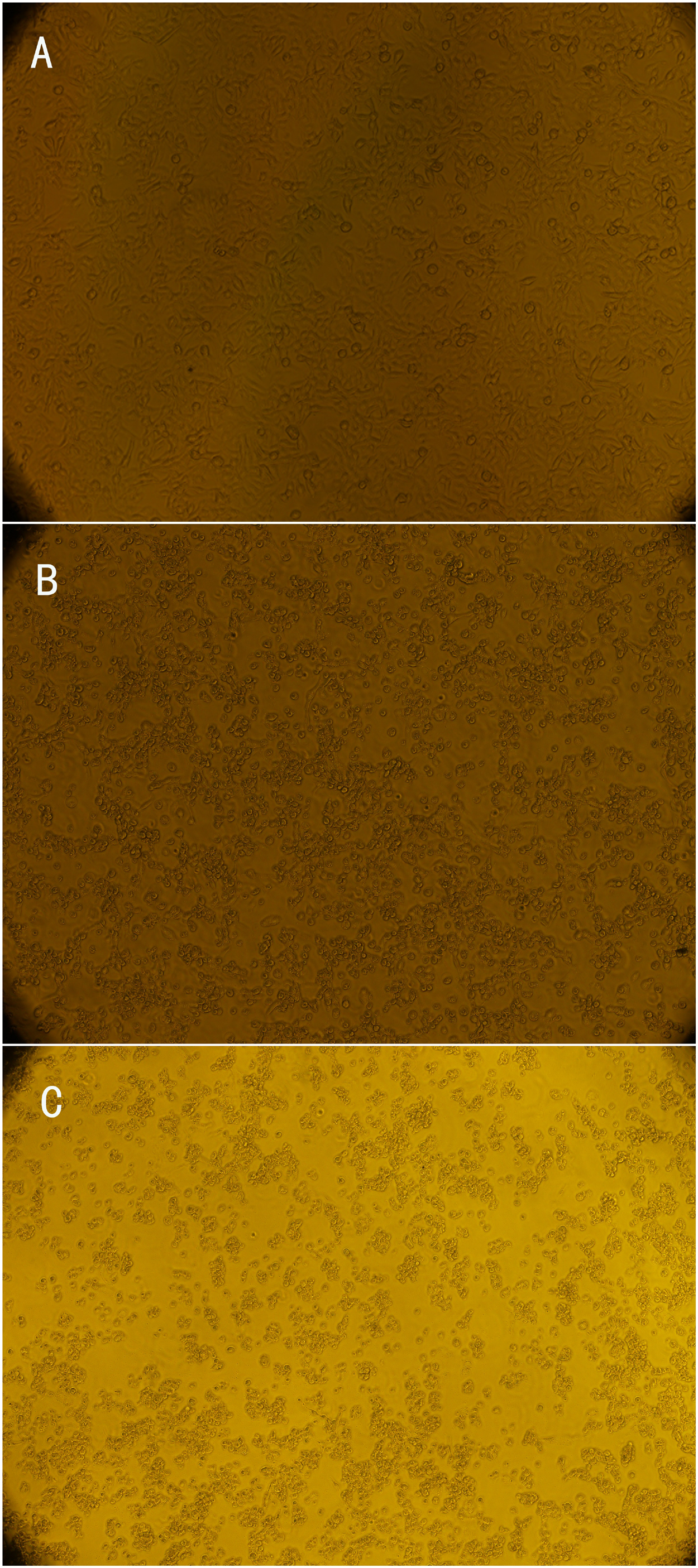

Supplement: Figure S1 — The results of viral isolation. (A) negative control. (B) CPE of EV71. (C) CPE of CoxA16. (TIF) [file pone.0042251.s001.tif]
